# Transcriptomic Signatures of Tacaribe Virus-Infected Jamaican Fruit Bats

**DOI:** 10.1128/mSphere.00245-17

**Published:** 2017-09-27

**Authors:** Diana L. Gerrard, Ann Hawkinson, Tyler Sherman, Cassandra M. Modahl, Gretchen Hume, Corey L. Campbell, Tony Schountz, Seth Frietze

**Affiliations:** a Department of Medical Laboratory and Radiation Sciences and Cell, Molecular, and Biomedical Sciences Program, University of Vermont, Burlington, Vermont, USA; b School of Biological Sciences, University of Northern Colorado, Greeley, Colorado, USA; c Department of Biological Sciences, Faculty of Science, National University of Singapore, Singapore; d Arthropod-Borne and Infectious Diseases Laboratory, Department of Microbiology, Immunology and Pathology, College of Veterinary Medicine and Biomedical Sciences, Colorado State University, Fort Collins, Colorado, USA; CDC

**Keywords:** arenavirus, bats, transcriptome, virus-host interactions

## Abstract

Tacaribe virus (TCRV) is a mammalian arenavirus that was first isolated from artibeus bats in the 1950s. Subsequent experimental infection of Jamaican fruit bats (*Artibeus jamaicensis*) caused a disease similar to that of naturally infected bats. Although substantial attention has focused on bats as reservoir hosts of viruses that cause human disease, little is known about the interactions between bats and their pathogens. We performed a transcriptome-wide study to illuminate the response of Jamaican fruit bats experimentally infected with TCRV. Differential gene expression analysis of multiple tissues revealed global and organ-specific responses associated with innate antiviral responses, including interferon alpha/beta and Toll-like receptor signaling, activation of complement cascades, and cytokine signaling, among others. Genes encoding proteins involved in adaptive immune responses, such as gamma interferon signaling and costimulation of T cells by the CD28 family, were also altered in response to TCRV infection. Immunoglobulin gene expression was also elevated in the spleens of infected bats, including IgG, IgA, and IgE isotypes. These results indicate an active innate and adaptive immune response to TCRV infection occurred but did not prevent fatal disease. This *de novo* assembly provides a high-throughput data set of the Jamaican fruit bat and its host response to TCRV infection, which remains a valuable tool to understand the molecular signatures involved in antiviral responses in bats.

**IMPORTANCE** As reservoir hosts of viruses associated with human disease, little is known about the interactions between bats and viruses. Using Jamaican fruit bats infected with Tacaribe virus (TCRV) as a model, we characterized the gene expression responses to infection in different tissues and identified pathways involved with the response to infection. This report is the most detailed gene discovery work in the species to date and the first to describe immune gene expression responses in bats during a pathogenic viral infection.

## Abstract (precis)

As reservoir hosts of viruses associated with human disease, little is known about the interactions between bats and viruses. Using Jamaican fruit bats infected with Tacaribe virus (TCRV) as a model, we characterized the gene expression responses to infection in different tissues and identified pathways involved with the response to infection. This report is the most detailed gene discovery work in the species to date and the first to describe immune gene expression responses in bats during a pathogenic viral infection.

## INTRODUCTION

Bats are a phylogenetically and geographically diverse group of mammals, with about 1,150 species (1, 2). Certain bat species have been identified as reservoir hosts of zoonotic viruses associated with significant human morbidity and mortality, including rabies virus and other lyssaviruses, Marburg virus, Nipah virus, and Hendra virus (3). They also are suspected reservoirs of other viruses, such as the ebolaviruses, and Middle East respiratory syndrome (MERS) and severe acute respiratory syndrome (SARS) coronaviruses (CoVs) (4–6). Each of these viruses can cause severe disease in humans but are not known to cause disease in their reservoir hosts (3, 7). Although nearly 200 viruses have been associated with bats, there are likely many more (8). As non-model organisms, virtually nothing is known about bat immune responses. Although bats appear to have small genomes relative to other mammals (9), genomic analyses suggest that bats share most features of other mammals (8, 10–12).

Despite serving as reservoir hosts of several zoonotic viruses, some bats are also susceptible to infectious diseases. White nose syndrome, which has caused the deaths of millions of bats in North America, is a fungal disease threatening some species with extinction (13–16). Bats can shed rabies virus and other lyssaviruses for prolonged periods, but the infection is always fatal (3, 17–21). Because bats are important members of their ecosystems, a better understanding of the immune responses and subsequent pathogenesis to infectious agents is essential. To this end, we developed a laboratory model for the study of infection of Jamaican fruit bats (*Artibeus jamaicensis*) by a natural bat pathogen, Tacaribe virus (TCRV) (11, 21, 22).

TCRV is a mammarenavirus first isolated from two species of diseased artibeus bats in the late 1950s near Port-of-Spain, Trinidad, and is most closely related to Junïn and Machupo viruses, which cause Argentine and Bolivian hemorrhagic fevers, respectively (23–25). Each arenavirus is associated with a specific host species, and the distribution of the host therefore dictates the distribution of the virus. All known reservoir hosts of mammarenaviruses are rodents; however, the reservoir host of TCRV remains unclear. It was suspected that artibeus bats were reservoirs of TCRV given its original isolation from multiple artibeus bats and the inability to detect it in other mammals (25–27). Interestingly, TCRV was isolated from lone star ticks collected in Florida in 2012 (28). The tick-derived isolate was nearly identical to the TCRV isolate from Trinidad (TRVL-11573), with 99.6% nucleotide identity across its genome (28). Recent studies by our group found that TCRV causes fatal disease or is cleared without pathology in Jamaican fruit bats, features that are inconsistent for a reservoir host (22). In many of these bats, substantial neutrophil and lymphocytic infiltration into tissues occurred, which suggests a role for these cells in the host response to TCRV (22).

The present study was designed to characterize the transcriptional responses of bats with TCRV disease. Accordingly, we performed RNA sequencing of spleens and liver and kidney samples from experimentally infected bats and generated a broad bat transcriptome rich in annotated genes. These target tissues were chosen because they represent the organs with the most significant pathology in our previous report (8). This report is the most comprehensive gene discovery work in the species to date and the first to describe immune gene expression responses in bats during an arenavirus infection.

## RESULTS

### High-quality *de novo* assembly and annotation of the Jamaican fruit bat transcriptome.

**FIG 1:**
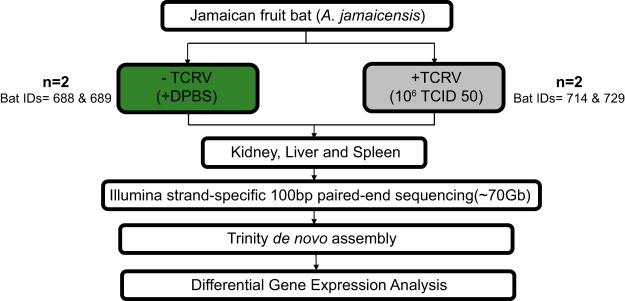
Transcriptomic analysis of Jamaican fruit bats infected with Tacaribe virus (TCRV). Jamaican fruit bats were inoculated with either TCRV or DPBS (*n =* 2 for each condition). *De novo* assembly of the Jamaican fruit bat transcriptome was performed using RNA-Seq data from kidney, liver, and spleen tissues. Differentially expressed genes were then identified in the uninfected and infected tissues using edgeR.

**FIG 2:**
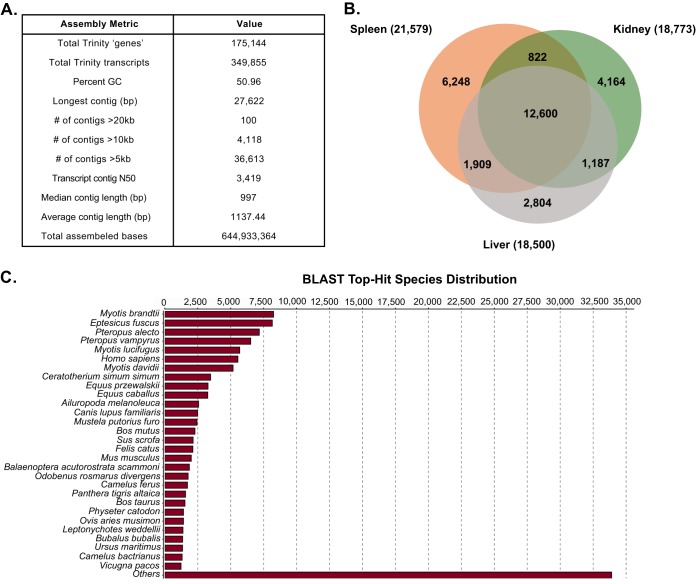
*De novo* assembly of the Jamaican fruit bat transcriptome. (A) Trinity assembly resulted in the construction of the *de novo*-assembled Jamaican fruit bat transcriptome with 644,933,364 assembled bases. (B) Examination of the identified contigs from the transcriptome assembly showed that 35% (12,600) are expressed in the spleen, kidney, and liver tissues (FRKM, >1). (C) We compared this transcriptome assembly to those of other mammals through BLASTX analysis and identified transcripts similar to those present in other bat species.

We previously reported a high mortality rate in Jamaican fruit bats experimentally infected with TCRV, in which high-dose inoculations (10^6^ 50% tissue culture infective doses [TCID_50_]) caused significant and fatal disease as early as 10 days postinfection (22). Histopathologic findings revealed multiple organ involvement in TCRV disease, including acute neutrophilic splenitis and white pulp hyperplasia, as well as plasmacytic and histiocytic splenitis. To profile the host pathogenic transcriptional response, we generated stranded poly(A)^+^ Illumina RNA-Seq (transcriptome sequencing) libraries using RNA extracted from the organs of experimentally infected bats. For this analysis, we harvested the livers, kidneys, and spleens from 2 control bats (Dulbecco’s phosphate-buffered saline [DPBS] treated) and 2 TCRV-infected bats with fatal disease (Fig. 1). Our previous analysis indicated TCRV RNA was present in each of these tissues at time of collection (22). A total of 12 pooled samples were sequenced, generating 693,106,150 raw 100-bp paired-end reads. After demultiplexing, trimming of poor-quality reads and adapter sequences, and removing duplicate reads, 691,108,820 nonredundant reads per sample were used for the transcriptome assembly. *De novo* assembly of the global transcriptome was performed using Trinity, resulting in 349,855 assembled transcripts of ≥300 bp (mean length of 997 bp) with an *N*_50_ of 3,419 bases that were clustered into 175,144 nonredundant clustered transcripts (unigenes) (Fig. 2A) (29). Inspection of these unigenes identified from the combined transcriptome showed that 35% of the contigs (12,600) are expressed in each of the three different tissues (fragments per kilobase per million [FPKM], >1), whereas the expression of many tissue-specific contigs was identified in the spleen, liver, and kidney (Fig. 2B).

The combined Jamaican fruit bat transcriptome was systematically annotated using the Trinotate pipeline, a software suite that automates the functional annotation of the assembled contigs (30). The annotation report for the combined assembly from the Trinotate pipeline (see Data Set S1 in the supplemental material) represents the predicted coding sequences of Jamaican fruit bat genes and the results of homology searches against the databases listed in Data Set S1. Among the 227,656 transcripts containing complete open reading frame (ORF) sequences, 124,204 nonredundant ORFs (54%) were associated with high-confidence coding predictions, BLAST homology and PFAM domain content (see Data Set S2 in the supplemental material). We compared this combined Jamaican fruit bat transcriptome assembly to those of other mammals through BLASTX analysis. The bat Brandt’s myotis (*Myotis brandtii*) had the highest number of related sequences (8,060 similar sequences [Data Set S2]). Among other mammals were the big brown bat (*Eptesicus fuscus*) and the black flying fox (*Pteropus alecto*) (with 7,947 and 6,955 similar sequences, respectively) (Fig. 2C).

10.1128/mSphere.00245-17.4 DATA SET S1  Artibeus transcriptome assembly Trinotate annotation report. Download DATA SET S1, XLSX file, 3 MB. Copyright © 2017 Gerrard et al. 2017 Gerrard et al. This content is distributed under the terms of the Creative Commons Attribution 4.0 International license.

10.1128/mSphere.00245-17.5 DATA SET S2  Top species distribution. Download DATA SET S2, XLSX file, 0.1 MB. Copyright © 2017 Gerrard et al. 2017 Gerrard et al. This content is distributed under the terms of the Creative Commons Attribution 4.0 International license.

### Differential gene expression following TCRV infection.

**FIG 3:**
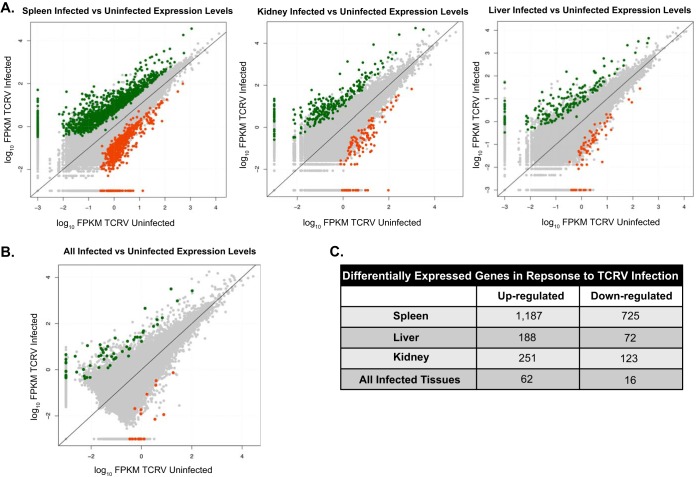
Differential gene expression analysis following TCRV infection in Jamaican fruit bats. We used a pairwise comparison of TCRV-infected samples against the corresponding control uninfected samples and found that the expression levels of hundreds of genes were altered with TCRV infection in the different tissues. (A) Differential expression analysis revealed upregulated genes (green) and downregulated genes (orange) defined by edgeR (log_2_ fold change of >2 and FDR of <0.01). (B) Inspection of altered genes in all infected tissues versus control tissues showed fewer changed genes common to all tissues. (C) Quantification of differentially expressed genes from panels A and B.

To investigate the molecular response of bats to TCRV infection, differential gene expression analysis was performed. We used a pairwise comparison of TCRV-infected samples against the corresponding controls and found that the expression levels of hundreds of different genes were altered during TCRV infection (Fig. 3A). The spleen had the largest number of differentially expressed genes (DEGs); among these 1,912 DEGs, 1,187 were upregulated and 725 were downregulated following infection (Fig. 3C; false discovery rate [FDR], <0.01; log_2_ fold change, >2). We also determined that the kidney and liver each had a greater number of upregulated genes (251 and 188, respectively) compared to the number of downregulated genes in these tissues following TCRV infection (123 and 72, respectively). A comparison of all TCRV-infected tissues against all of the uninfected controls revealed 62 upregulated and 16 downregulated genes (Fig. 3B and C).

### Immune gene expression profile in response to TCRV infection.

**FIG 4:**
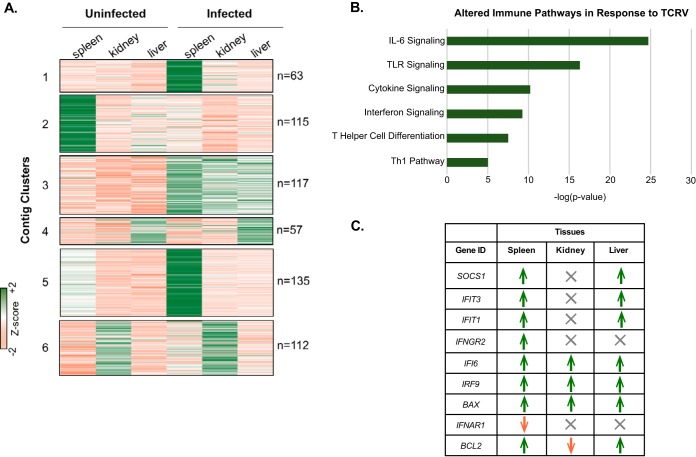
Immune-specific expression analysis of TCRV-infected *Artibeus jamaicensis* bats. (A) Immport database immune-related genes and their expression values (FPKM) were clustered (*k*-means = 6) to investigate the relationship between uninfected and infected tissues. (B) We performed Ingenuity Pathway Analysis to characterize the specific immune pathways for those genes identified in cluster 3 (*n =* 117) from panel A. (C) Interferon signaling was among the top pathways identified to be altered after TCRV infection. We then identified these specific genes involved in interferon signaling and explored their alterations in the different tissues in response to TCRV infection. Green corresponds to upregulated and orange to downregulated (FDR, <0.01; log_2_ fold change, >2); gray indicates no significant differential expression.

To gain specific insight into the immune-related gene expression altered in response to TCRV infection, we utilized the ImmPort database to identify those TCRV-altered genes that relate to immune-system functions (31). Approximately 23% of the 4,723 genes available in the database corresponded to the differentially expressed genes annotated in our analysis (see Data Set S3 in the supplemental material). The coordinating transcript expression values of these identified immune genes were used to evaluate the relationship between the specific uninfected and infected tissues (Fig. 4A). While all three tissue types studied had unique expression profiles in the infected samples, we further analyzed the transcripts contained in cluster 3, which represent sequences with overall shared expression patterns and found that these corresponding genes map to pathways identified to be affected in response to viral infection (Fig. 4B). Notably, with the use of Ingenuity Pathway Analysis (IPA [Qiagen]), we identified the interferon (IFN) signaling pathway to be among the top pathways altered upon TCRV infection (cluster 3, IPA [see Data S4 in the supplemental material]). IFNs are a family of cytokines secreted by host cells in response to viruses and other pathogens to confer antiviral states upon uninfected neighboring cells in an effort to prevent spread of infection (32). Given that the IFN response has been explored in bats in regard to pathogen-host response (33), we then further examined the relationship between these factors within the spleen, kidney, and liver in response to TCRV infection and found that while most of the identified IFN pathway-related genes were upregulated, all of the factors identified in this pathway had statistically significant differential expression (log_2_ fold change, >2; FDR, <0.01) in the spleen (Fig. 4C; see Fig. S1 in the supplemental material). We validated differential expression of select immune genes via reverse transcription-quantitative PCR (RT-qPCR) and confirmed upregulation of *ISG15* and *IRF7* in the spleen and kidney tissues and downregulation of *HLA-DRA* in the kidney (see Fig. S2 in the supplemental material).

10.1128/mSphere.00245-17.1 FIG S1  Interferon alpha/beta signaling pathway. Reactome pathway of all upregulated genes in response to TCRV infection in Jamaican fruit bats. (Items containing upregulated genes are indicated in purple.) Download FIG S1, EPS file, 1.9 MB. Copyright © 2017 Gerrard et al. 2017 Gerrard et al. This content is distributed under the terms of the Creative Commons Attribution 4.0 International license.

10.1128/mSphere.00245-17.2 FIG S2  RNA-Seq validation using RT-qPCR. (A) Primers designed against immune-related genes (see Materials and Methods). (B) RNA-Seq and RT-qPCR expression levels in TCRV-infected tissues relative to β-actin. Download FIG S2, EPS file, 1.7 MB. Copyright © 2017 Gerrard et al. 2017 Gerrard et al. This content is distributed under the terms of the Creative Commons Attribution 4.0 International license.

10.1128/mSphere.00245-17.6 DATA SET S3  Annotated (DEG) gene list for all treatments. Download DATA SET S3, XLSX file, 0.1 MB. Copyright © 2017 Gerrard et al. 2017 Gerrard et al. This content is distributed under the terms of the Creative Commons Attribution 4.0 International license.

10.1128/mSphere.00245-17.7 DATA SET S4  Ontology analysis of Tacaribe virus-infected Jamaican fruit bats. Download DATA SET S4, XLSX file, 0.1 MB. Copyright © 2017 Gerrard et al. 2017 Gerrard et al. This content is distributed under the terms of the Creative Commons Attribution 4.0 International license.

In addition to the IFN-signaling pathway, we identified signaling pathways for Toll-like receptors (TLRs) and interleukin-6 (IL-6) (among other cytokines), as well as pathways for T-helper cell differentiation and the Th1 pathway (Fig. 4B; see Fig. S3 and Data Set S4 in the supplemental material). Further analysis of all DEGs via the Reactome plugin (Cytoscape) identified additional key pathways involved in the immune response (Table 1; Data Set S4, “All up-regulated Reactome”). Specifically, we identified increased transcript levels of several cytokine genes (*IL6*, *IL8*, *IL1A*, *IL1B*, and *IFNG*) and chemokine genes (*CXCL1*, *CXCL2*, *CXCL3*, *CXCL5*, and *CXCL6*). To highlight markers associated with circulating immune cells, we focused on those DEGs that were common to two or more tissues, and the data are consistent with increased infiltration of neutrophils into the infected tissues. In kidneys, neutrophil infiltration can cause hyperinflammation and kidney damage (34). This is further supported by the presence of enriched expression levels of neutrophil gelatinase-associated lipocalin (*NGALl*) in all three tissues, which is a biomarker for renal damage in humans (35).

10.1128/mSphere.00245-17.3 FIG S3  Reactome pathway analysis of TCRV-infected Jamaican fruit bats. (A) We identified key pathways involved in the immune response via Reactome pathway analysis of differentially expressed genes in TCRV-uninfected and -infected tissues. (B) We then further explored Reactome pathways associated with infected tissues. Download FIG S3, EPS file, 3.3 MB. Copyright © 2017 Gerrard et al. 2017 Gerrard et al. This content is distributed under the terms of the Creative Commons Attribution 4.0 International license.

**TABLE 1:** Immune-related Reactome pathways upregulated during TCRV infection

Reactome pathway^a^	Spleen		Kidney		Liver	
	*P* value	FDR	*P* value	FDR	*P* value	FDR
Cytokine signaling						
Cytokine signaling in immune system	0.000	<8.3e−05	0.000	<3.3e−04	0.000	<3.3e−04
IFN signaling	0.000	<1.0e−0.04	0.000	<2.0e−04	0.000	<5.0e−04
IFN-α/β signaling	0.000	<2.0e−0.04	0.000	<5.0e−04	0.000	<5.0e−04
IFN-γ signaling	0.000	<9.1e−05	0.000	<1.0e−03	0.000	<2.5e−04
Chemokine receptors bind chemokines	0.001	0.001	0.028	0.235	0.003	0.044
Innate immune response						
TLR cascades	0.005	0.009	0.000	<2.5e−04	0.081	0.615
MyD88-independent cascade	0.014	0.036	0.000	0.002	0.077	0.600
Cytosolic sensors of pathogen-associated DNA	0.32	0.963	0.000	0.002	0.027	0.248
Factors involved in megakaryocyte development and platelet production	0.001	0.001	0.084	0.458	0.581	1.000
IL-1 signaling	0.058	0.151	0.051	0.367	0.502	1.000
DAP 12 interactions	0.301	0.915	0.284	0.967	0.080	0.615
Hemostasis						
Degradation of extracellular matrix	0.000	<5.3e−05	0.000	0.001	0.000	0.000
Extracellular matrix organization	0.009	0.018	0.000	0.001	0.000	0.005
Oxidative stress-induced senescence	0.004	0.006	0.735	1.000	0.673	1.000
Formation of fibrin clot (clotting cascade)	0.020	0.048			0.000	0.003
Dissolution of fibrin clot	0.055	0.140	0.187	0.703	0.013	0.123
Hemostasis	0.022	0.052	0.214	0.790	0.011	0.104
Adaptive immune response						
Class I MHC-mediated antigen processing and presentation	0.778	1.000	0.846	1.000	0.005	0.082
Fc receptor signaling	0.915	1.000	0.497	1.000	0.921	1.000

a IFN, interferon; TLR, Toll-like receptor; IL-1, interleukin-1; MHC, major histocompatibility complex.

**FIG 5:**
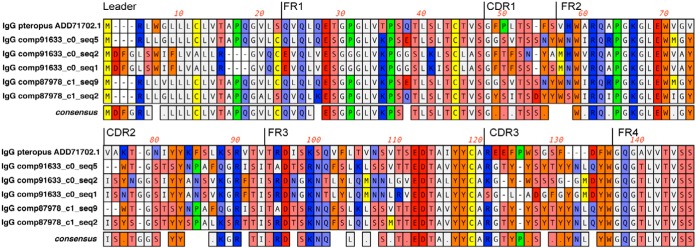
Amino acid alignment of Jamaican fruit bat IgG V regions. Five V region transcripts were identified in the Jamaican fruit bat spleen transcriptomes and aligned with a V region of an annotated black flying fox V region (50). Framework regions (FR1 to FR4) and complementarity-determining regions (CDR1 to CDR3) were identified and exhibited differences between each transcript.

Transcripts for IgG, IgM, IgA, and IgE were identified in the spleen data, and the level of each was significantly elevated in the infected bats. Six transcripts of IgG heavy chains were identified, including 5 with complete and distinct V regions. The six IgG constant regions were identical, other than one that contained a Thr in place of an Ala, which could represent an allele or a sequencing error. The hinge regions, which are frequently different between IgG subclasses within a species, were identical in all 6 transcripts. These two features suggest that Jamaican fruit bats have a single IgG isotype. The 5 V regions contain the canonical mammalian Ig sequences, including 4 framework regions (FR) and 3 complementarity-determining regions (CDRs) (Fig. 5). Three distinct CDR3 sequences were present in these 5 transcripts. Two had 2 tyrosine residues, whereas the three that were identical had 6, substantially more than what has been reported in the CDR3s of other bat species (36, 37). Sequences for T-cell receptor alpha (TCR-α), TCR-β, TCR-γ, and TCR-δ constant region domains were present in the assembly, although none had complete V regions (data not shown). Expression of TCR-α was identified in all uninfected and infected tissues, TCR-β in all spleen and liver tissues, TCR-γ in all spleen and liver tissues, and TCR-δ in all spleen and uninfected liver tissues.

### Gene ontology of annotated differentially expressed genes in TCRV-infected tissues.

To characterize the overall transcriptome in response to TCRV infection, we performed an unbiased evaluation of the top 10 Reactome pathways (ranked by *P* value) associated with DEGs in various organs (Table 2; Fig. S2 and Data Set S4, “Top Pathways Reactome”). In all three tissues, genes controlling cell cycle progression were elevated, including many associated with hypoxia, cell stress, senescence, and chromatin organization.

**TABLE 2:** Top reactome pathways depicting up- or downregulated genes

Reactome pathway	*P* value	FDR	Treatment condition^a^
Defective CYP21A2 causes adrenal hyperplasia 3 (AH3)	0.000	0.000	Kidney, −
Defective CYP11B1 causes adrenal hyperplasia 4 (AH4)	0.000	0.000	Kidney, −
Phase 1—functionalization of compounds	0.000	0.000	Kidney, −
Defective CYP7B1 causes spastic paraplegia 5A autosomal recessive (SPG5A) and congenital bile acid synthesis defect 3 (CBAS3)	0.000	0.000	Kidney, −
Collagen degradation	0.000	0.000	Liver, +
Organic cation transport	0.000	0.000	Kidney, −
Collagen biosynthesis and modifying enzymes	0.000	0.000	Kidney, −
NCAM1 interactions	0.000	0.000	Kidney, −
Degradation of extracellular matrix	0.000	0.000	Liver, +
Chemokine receptors bind chemokines	0.000	0.001	Spleen, −

a −, untreated; +, treated.

Spleen differential expression analysis indicated that immune system pathways were significantly elevated, including type I and II IFN signaling, antiviral IFN-stimulated genes (ISGs), interleukin signaling, and T and B cell activation pathways. Interestingly, genes involved in the complement cascade were repressed, including the genes for phosphatidylinositol 3-kinase and complement receptor 1. *SH2B1*, a gene encoding an important signal transduction adaptor in several pathways, including JAK, prolactin, platelet-derived growth factor, and nerve growth factor signaling, was also significantly downregulated in the spleen (38). We also identified repression of proapoptotic genes *BMP* and *PMAIP1* as well as repression of genes involved in calcium mobilization following TCRV infection (39).

In the liver, Reactome analysis revealed strong immune activation signatures, including T-cell receptor and CD28 costimulatory signaling. No evidence of B-cell or NK cell activities was present. TLR and RIG-I/MDA5 signaling for type I IFN responses was also elevated, despite no evidence of differentially expressed type I IFN genes. Unlike the spleen, complement pathways were also enriched. The IFN-γ signaling pathway was also identified, although *IFNG* itself was not differentially expressed. Despite these findings, further indications of apoptotic activation were not differentially expressed in the livers. As in the spleen, many metabolic genes were elevated, whereas genes involved in calcium mobilization were repressed.

In the kidneys, Reactome analysis suggested that platelet calcium-associated degranulation may occur; the genes *F13A1* and *TMSB4X* were elevated along with other genes involved in calcium mobilization. As in the other tissues, evidence of innate antiviral responses was present, including TLR signaling, RIG-I/MDA5 activity, and type I IFN signaling genes. Interleukin and IFN-γ signaling were also among the pathways characterized secondary to gene upregulation in the kidneys.

## DISCUSSION

Our previous work demonstrated that TCRV is pathogenic to Jamaican fruit bats and has allowed us to perform the most complete examination to date of a pathogenic virus infection in a bat species (22). Despite their importance to human health as reservoirs of emerging viruses, the characterization of infections in bats at the cellular and molecular levels has been limited relative to other model organisms, such as rodents. Fortunately, the emerging advantage of next-generation sequencing technologies has been fundamental to our understanding of disease responses; however, minimal reference data sets are available for bats. To this end, our group was among the first to perform next-generation sequencing on bats with a small-scale Illumina sequencing of kidney and lung tissues in a single library from the Jamaican fruit bat (8, 11). Furthermore, in the present study we generated a high-quality transcriptomic data set for the Jamaican fruit bat and comprehensively profiled the altered immune genes in response to TCRV infection.

To gain insight into the pathogenic infection of Jamaican fruit bats, we performed high-throughput RNA sequencing of TCRV-infected spleen, liver, and kidney tissues and corresponding sham-inoculated controls. We produced high-quality nonredundant reads, and our Trinity *de novo* assembly resulted in 349,855 transcripts, which were further assembled into 124,204 contigs. The number of nonredundant contigs we identified is similar to those from other transcriptome assemblies reported for the black flying fox (126,378) (10), Rickett’s big-footed bat (104,987), and the greater short-nosed fruit bat (171,394) (40).

We employed a pairwise comparison of all infected tissues versus sham-inoculated controls to identify altered gene expression levels upon pathogenic TCRV infection. We utilized a log_2_ fold change cutoff of >2 with an FDR of <0.01. We chose a stringent cutoff because of our small sample size (*n =* 2) for each tissue type under each condition. This revealed approximately 25% more genes upregulated than downregulated. The spleen is instrumental in systemic and local immune responses and has been used to study viral responses in many organisms, including bats (3, 41). We observed the greatest number of differentially expressed genes in spleen tissues compared to the liver and kidneys. Further analysis revealed that the majority of these differentially expressed genes identified in the spleen belonged to immune-related pathways.

Ingenuity Pathway Analysis identified enrichment of the helper T cell differentiation and Th1 pathway (Fig. 4B) genes* IFNG*, *IFNGR2*, *IL12RB2*, *IL6ST*, *SOCS1*, and *SOCS2*, supporting a role for mobilization of a Th1 response. Despite this, levels of helper T cell genes, such as CD4 or T-cell receptor (TCR) genes, were not statistically different in infected bats. CD4 sequences were not in the assembly, suggesting the CD4 level was below the threshold of depth of RNA-Seq. TCR-α, TCR-β, TCR-γ, and TCR-δ sequences were present in the assembly, and they appear to share features found in TCRs of other species. There was insufficient sequence data to evaluate TCR variable, diversity, or joining segments for T-cell receptors. Further studies using next-generation repertoire sequencing will be required to fully examine the TCR loci. Unfortunately, without monoclonal antibodies to identify CD4^+^ or CD8^+^ cells by flow cytometry, it is difficult to determine whether T cells are expanding in response to infection. Other indicators of T-cell activation include the elevated expression of granzyme A and B genes (*GZMA*, *GZMB*), *IL-12* and *CCL5* (*RANTES*), and the activated T-cell chemotactic factor gene *CXCL1* in the spleens of infected bats.

Transcripts for IgG, IgM, IgA, and, interestingly, IgE were significantly higher in the infected bats. IgE is not typically associated with viral infections, but has been associated with anaphylaxis after influenza vaccinations (42–44). No transcripts for IgD were present in the transcriptome, similar to what has been observed for other microbats (45). Alignments of the 6 IgG transcripts were identical, except for one transcript that had a Thr instead of Ala at position 395, which likely represents an allele or sequencing error. Only one IgG transcript has been found in Seba’s fruit bat (*Carollia perspicillata*) (45); thus, it is not unexpected that Jamaican fruit bats may only have a single IgG isotype. The Jamaican fruit bat IgG shares 94% identity and 96% similarity with the Seba’s fruit bat IgG constant region. The hinge regions of all IgG transcripts were also identical and distinct from those of Seba’s fruit bat IgG. Hinge regions are generally considered hallmark indicators of IgG subclasses (46). It is possible that Jamaican fruit bats have IgG subclasses but without a genome or transcriptome profiling of Ig transcripts this question could be difficult to address.

The heavy-chain variable regions of the 5 Jamaican fruit bat transcripts showed many differences, suggesting they represent distinct segments and multiple V region gene families. The limited number of V regions makes it difficult to assign Jamaican fruit bat sequences to gene families. We are unable to estimate the number of V, D, or J segments with the transcriptome data; however, bats appear to have much larger numbers of these segments than most mammals (36, 37). It is noteworthy that the three CDR3 regions have more tyrosine residues than are found in most other bat species immunoglobulins. The presence of tyrosines is thought to contribute to antibody interactions with a spectrum of epitopes (47–49), and the lack of these in bat antibodies has been postulated to account for why bats have generally poorer responses to infectious agents (50, 51).

Only a single variable region light-chain sequence was significantly elevated in the infected bats, which had most similarity to the IgLV7 variable gene family. Studies of big brown bats (*Eptisicus fuscus*) suggest they express predominantly, if not exclusively, λ light chains; thus our findings are similar (52). Considering that a single light chain was elevated in infected bats, it may be possible to clone this cDNA and coexpress it with each of the 5 heavy-chain sequences described herein to determine if the antibodies are reactive to TCRV antigens.

We also detected elevated expression of polymeric immunoglobulin receptor (*PIGR*), which exports IgA antibodies across the epithelium into mucosa (53), in the spleens of infected bats. Considering the presence of TCRV in oral and rectal swabs and in the lungs (22), it is likely that virus-specific IgA is present at these sites. The development of antibodies to artibeus IgA will be necessary to verify this.

The principal gene for somatic hypermutation (SHM) that leads to affinity maturation is activation-induced cytidine deaminase (*AID*) (54); however, despite its presence in all four bats in this study, its expression was not significantly elevated in the spleens. Other genes involved in SHM (54) were elevated, including those coding for DNA polymerase θ (*POLQ*), polymerase η (*POLN*), and replication protein A (*RPA*). The level of *APOBEC3*, coding for another RNA-editing enzyme with lower SHM activity (55), was not elevated. Examination of little brown bat (*Myotis lucifugus*) antibody cDNA sequences suggests bats do not use SHM to a great extent (36), and our findings are congruent with this observation. In our previous work with TCRV and MERS coronavirus (CoV) infection of Jamaican fruit bats (22, 56), antibody responses were poor, suggesting that affinity maturation is limited in bats.

Global differential expression evaluation of TCRV-infected tissues revealed alterations in calcium mobilization, a characteristic mechanism of host response to infection by viruses, including arenaviruses (57). Additionally, our analysis revealed few indications of NK cell activation and minimal expression of genes that are associated with T-cell exhaustion (i.e., *Ly6e* and *Fcgr3*). It is noteworthy that bats appear to be missing many NK cell-associated genes (10, 58, 59); thus it may be that the functions of bat NK cells are substantially different from those of human or mouse NK cells. We detected increased *IFNG*, *GZMA*, and *GZMB* expression in the spleen infected tissues, and while these proteins are produced by both NK cells and T cells, we believe their presence correlates more strongly with a T-cell origin due to the increased number of T-cell-associated genes upregulated relative to NK cell genes. Moreover, the bats in this report were euthanized on days 10 and 11, a time point at which T-cell activation should be occurring. Thus, T-cell exhaustion, a feature of some lymphocytic choriomeningitis virus (LCMV) isolates, is likely not occurring in bats infected with TCRV (60, 61).

We identified several genes associated with neutrophil activation. These results are consistent with our previous histopathological findings in this species, where we noted neutrophilic infiltration that was likely a result of proliferating lymphocytes (22). Additionally, our results are also consistent with a recent Lassa virus isolate from Mali that similarly induces neutrophil infiltration in nonhuman primates (62). The abundant expression of *NGAL* may provide a diagnostic tool; its protein, neutrophil gelatinase-associated lipocalin, is secreted in the urine, which is detectable with commercially available diagnostic kits (e.g., Pacific Biomarkers, Seattle, WA).

A recent study looked at differential gene expression in an embryonic cell line from Egyptian fruit bats infected with Marburg virus (63). In contrast to the observed host responses in the Egyptian fruit bat cells, we identified the JAK/STAT signaling pathway as one of the immune-related pathways upregulated in response to pathogenic TCRV infection, suggesting a contributory role for this pathway in pathogenesis. Additionally, a study exploring the innate immune response to Newcastle disease virus in large flying fox cells, a newly characterized a subset of antiviral factors was found (64). Among these factors was the *CHAC1* gene, which we identified to be 4-fold upregulated in spleen and kidney tissues. Together, this evidence along with our previous pathogenicity studies shows that a typical antiviral response occurs to TCRV in Jamaican fruit bats.

We focused additional analyses on immune genes with similar expression in all tissues (cluster 3 [Fig. 4A]). Among the top pathways identified was the IFN signaling pathway. The signaling factors in this pathway exert their antiviral activities through the induction of other antiviral proteins (32). The IFN response has been explored in bat cells (33), and in all bat species examined, the type I IFN locus has undergone substantial contraction, with only three functional IFN-α genes but with constitutive IFN-α expression in at least one species (65). Specifically, recent discoveries have revealed enhanced IFN signaling in antiviral immunity and have identified its involvement in arenavirus response mechanisms. We therefore furthered our analysis regarding these pathways (51, 66). We found that most IFN signaling genes identified in this subset were upregulated in all tissues; however, 5 of these genes had no significant differential expression identified in the kidney and 2 had none in the liver. Notably, of the differentially expressed factors, the *IFNAR1* gene was downregulated in the spleen and *BCL2* was downregulated in the kidney. Apoptotic pathways play a critical role as defense mechanisms for a host when infected by a viral pathogen; *BCL2* encodes an antiapoptotic protein that is known to be involved in a typical antiviral response (67), and the observed downregulation of *BCL2* in the kidneys upon TCRV infection suggests promotion of apoptotic pathways stimulated by IFN signaling in response to infection. In contrast, *BCL2* was determined to be upregulated in spleen and liver tissues. Additionally, another antiapoptotic factor gene, *Mcl-1*, was also upregulated in these tissues. Recent work with mice infected with LCMV, as well as other studies, has demonstrated the involvement of these factors in promoting naive T-cell survival and memory T cell activation (68, 69). Together, these results support congruency of our annotated transcriptome given what is known about the coordination of immune genes altered in response to viral infection as well as the identification of genes specific to the antiviral response in bats (61).

As might be expected during an acute antiviral response, IFN-stimulated gene 15 (*ISG15*) was elevated in infected tissues. Reactome pathway analysis identified ISG15 in several immune pathways, including the innate immune response, cytokine signaling, IFN-α/β signaling, and RIG-I/MDA5-mediated induction of IFN-α/β pathways, which has also been previously identified in a bat pathogenic viral response (64). *ISG15* is an important gene in the innate immune response, particularly the type I IFN antiviral response; however, the *ISG15*-encoded protein has recently been demonstrated to have additional functions as a ubiquitin-like modifier that covalently conjugates to other cellular proteins to form an “ISGylated” complex (70). Various roles of ISG15 have been identified in immune responses; when secreted extracellularly, ISG15 can act to drive expression of IFN-γ, which was elevated in the spleen. Alternatively, intracellular expression can modulate type I IFN signaling (71).

Although *IFN*-α, *IFNB*, *IFNL*, and *IFNG* transcripts were present in all of the tissues analyzed, the only differentially expressed transcript was *IFNG* in the spleen. In contrast, indications of downstream signaling initiated by IFN type I and type II were present, suggesting either transcript turnover prior to the time of sample collections or the potential for alternative routes of pathway activation. Previous work examining *in vitro* infection of the black flying fox with Tioman virus suggests a prominent role for IFN-γ (72). To this end, there are potential differences between bat species in terms of their responses to viruses that may account for apathogenic infections (e.g., reservoir hosts) or disease. Future work with cell culture from our model system may help to clarify these points. Furthermore, the DEGs involved in IFN-α/β signaling suggest that a typical antiviral innate immune response occurred in the bats. Within the spleen, expression of 36% of the genes was upregulated in the IFN-α/β signaling pathway, whereas the kidneys and livers had approximately 16% and 34% elevated expression of these same genes, respectively. This indicates a more robust type I IFN response in the spleen.

Together, *de novo* transcriptome analysis of our high-throughput RNA-Seq data from Jamaican fruit bats infected with TCRV provides a high-quality data set and also a comprehensive gene expression analysis of immune gene expression responses in bats during a pathogenic infection. This data set will provide a strong basis for additional analyses. Further investigation of our identified pathways *in vitro* and *in vivo* will significantly contribute to our understanding of pathogenic viral infections in bats. Moreover, the data here will facilitate future experimental studies of artibeus bats and their cells, which have been used as models for MERS CoV and Zaire Ebola virus and which are suspected reservoirs of the recently discovered bat influenza viruses (56, 73, 74).

### Conclusion.

This study provides a comprehensive analysis of the transcriptional landscape of Jamaican fruit bats during infection with Tacaribe virus. This natural pathogen of artibeus bats causes high-mortality disease with similar clinical manifestations to the South American hemorrhagic fevers and Lassa fever. In summary, this analysis identified the global response to TCRV infection. Our results suggest diverse immune responses, including alterations in neutrophil activation, interferon signaling, markers for lymphocytes, and antibodies. We found substantial signatures of neutrophil activation in the spleen, kidney, and liver of bats with fatal disease. The innate and adaptive immune response appeared to be functional and typical of the canonical antiviral response. Many activation markers of T and B lymphocytes were also found; however, few indications of NK cell activity or T-cell exhaustion were apparent. IgG, IgM, IgA, and IgE sequences were abundantly expressed in the spleens of infected bats, and five immunoglobulin heavy-chain V segments were identified. Despite the clear evidence of antibody synthesis during infection, AID expression was not elevated, suggesting somatic hypermutation and affinity maturation were absent or minimal. Analysis of immunoglobulin heavy-chain and TCR V regions suggests that Jamaican fruit bats have canonical immunoglobulin and TCR genes found in most mammals. Moreover, the species appears to have a single IgG subclass. These results are the most extensive gene discovery work completed in Jamaican fruit bats to date and the first to describe differential immune gene expression in bats during a pathogenic virus infection.

## MATERIALS AND METHODS

### Experimental TCRV infection in bats, sample collection, and RNA extraction.

Experimental infections of Jamaican fruit bats were previously reported (22). Briefly, two Jamaican fruit bats were inoculated with 100 μl of sterile Dulbecco’s phosphate-buffered saline (DPBS) as negative controls (bat IDs 688 and 689), and two bats were inoculated with 100 μl containing 10^6^ TCID_50_ TCRV (bat IDs 714 and 729). Negative control bats were euthanized at the end of the experimental period (45 days), whereas TCRV-infected bats were euthanized as they became moribund (days 11 and 18, respectively). Necropsies were performed directly following euthanasia, and organs were harvested and collected in RNAlater stabilization reagent (Qiagen). RNA was extracted from flash-frozen tissues by homogenization with a Mini Bead Beater (BioSpec Products, Inc.), using QiaShredder columns with the RNeasy kit (Qiagen).

### RNA-Seq.

Stranded Illumina libraries for each tissue were prepared from total RNA using the NEB Ultra Directional RNA library prep kit with poly(A) selection. Sequencing (paired-end 100 bp) was performed on the Illumina HiSeq-2000 platform at the UC Denver Genomics core.

### Read processing and assembly.

For transcriptome assembly, raw reads were filtered for adapter sequences and low-quality reads, and assembly was performed using Trinity (30) with the following parameters: —min_contig_length 300—min_glue 3—min_kmer_cov 2. Resulting contigs were processed for read alignment and abundance estimation with Bowtie and RSEM (75, 76). Differential expression was performed using the edgeR package within the Trinity differential analysis pipeline using default parameters (77). A pairwise comparison was made between TCRV-infected samples and control uninfected samples. Genes were considered differentially expressed with an FDR of <0.01 and a log_2_ fold change of >2. The complete list of annotated differentially regulated genes in each organ can be found in Data Set S3.

### Gene ontology and pathway analysis.

BLAST alignments and functional annotations were performed using Blast2GO Pro or Ingenuity Pathway Analysis (78, 79). Specific parameters can be found in Data Set S5 in the supplemental material, and the resulting outputs are summarized in Data S4. Direct pathway analysis for immune-related genes was performed using the gene list from the ImmPort database (31).

10.1128/mSphere.00245-17.8 DATA SET S5  BLAST2GO Pro parameters used. Download DATA SET S5, XLSX file, 0.1 MB. Copyright © 2017 Gerrard et al. 2017 Gerrard et al. This content is distributed under the terms of the Creative Commons Attribution 4.0 International license.

### Immunoglobulin sequence analysis.

Contigs for immunoglobulins were translated using the default translation table of MacVector software. MUSCLE alignments were made to identify leader, framework regions, and complementarity-determining regions of the V segments using a black flying fox sequence as a reference (NCBI GenBank accession no. ADD71702.1) (50). Heavy chains and hinge regions were identified by BLAST against other *Chiroptera*.

### RT-qPCR validation of RNA-Seq data.

The experimental primer sequences used in RT-qPCR analysis are listed in Fig. S2. cDNA was generated using SuperScript III reverse transcriptase (Thermo Scientific) and SYBR Select master mix for CFX (Applied Biosystems). The same two Jamaican fruit bats that were used for uninfected samples in RNA-Seq were also used as uninfected samples for RT-qPCR.

### Accession number(s).

Raw reads have been deposited into GenBank under GenBank accession no. GSE75771.
